# Structure and Genome Organization of *Cherry Virus A* (*Capillovirus, Betaflexiviridae*) from China Using Small RNA Sequencing

**DOI:** 10.1128/genomeA.00364-16

**Published:** 2016-05-12

**Authors:** Jiawei Wang, Ying Zhai, Weizhen Liu, Amit Dhingra, Hanu R. Pappu, Qingzhong Liu

**Affiliations:** a Key Laboratory for Fruit Biotechnology Breeding of Shandong Province, Shandong Institute of Pomology, Taian, Shandong, China; b Department of Plant Pathology, Washington State University, Pullman, Washington, USA; c Department of Crop and Soil Sciences, Washington State University, Pullman, Washington, USA; d Department of Horticulture, Washington State University, Pullman, Washington, USA

## Abstract

*Cherry virus* A (CVA) (*Capillovirus, Betaflexiviridae*) is widely present in cherry-growing areas. We obtained the complete genome of a CVA isolate (CVA-TA) using small RNA deep sequencing, followed by overlapping reverse transcription-PCR (RT-PCR) and rapid amplification of cDNA ends (RACE). The newly identified 5′-untranslated region (5′-UTR) from CVA-TA may form additional hairpin and loop structures to stabilize the CVA genome.

## GENOME ANNOUNCEMENT

Cherry virus A (CVA) belongs to the genus *Capillovirus*, family *Betaflexiviridae*. It was first reported in 1995 from *Little cherry virus 1*-infected sweet cherry (1). The genomes of capilloviruses consist of two open reading frames (ORFs). ORF1 encodes a 226-kDa fusion protein, which includes the RNA-dependent RNA polymerase (RdRp) and the coat protein (CP), and ORF2, located within the ORF1 but with a different reading frame, encodes the putative movement protein (MP) (1).

During 2011 and 2013, a survey was carried out for viruses on infected sweet cherry (*Prunus avium* L.) trees of the cultivar “Red Lamp” in Shandong Province, which is the most important sweet cherry-producing area in China. We obtained a Chinese CVA isolate and named it CVA-TA. The complete genome of CVA-TA was determined using small RNA deep sequencing, followed by overlapping reverse transcription-PCR (RT-PCR) and rapid amplification of cDNA ends (RACE).

Total RNA was extracted from CVA-TA-infected leaves in three biological replicates and then sequenced via the Illumina HiSeq 2000 platform. Contigs were assembled using CLC Genomics Workbench 7.5 (CLC bio, Aarhus, Denmark). The complete genome sequence of CVA-TA was cloned by overlapping RT-PCR. 5′ and 3′ terminal sequences were cloned by RACE. The resulting overlapping and RACE amplicons were cloned into the pEASY-T1 vector and sequenced. The sequences were assembled using the software Vector NTI 10.3 (Invitrogen, Carlsbad, CA, USA). The secondary structures of 5′-untranslated regions (5′-UTRs) from CVA isolates were predicted using MFOLDROOT (2).

Small RNA sequencing generated >2,000,000 raw reads from each of the three samples. A total of 15 Mbp in length of high-quality reads were obtained after trimming the adaptor and removing low-quality reads and reads <18 nucleotides. After *in-silico* removal of the host small RNAs, contigs at least 100 bp long were assembled. Based on the result of small RNA sequencing, the complete genome sequence of CVA-TA was amplified by overlapping RT-PCR, 5′-RACE, and 3′-RACE. A total of 7,434 nucleotides (nt) of the viral genome were obtained as cDNA, cloned, and sequenced. The CVA-TA genome consists of a 106-nt 5′-UTR, 302-nt 3′-UTR, and two large ORFs, ORF1 (7,026 bp, from positions 107 to 7132) and ORF2 (1,389 bp, from positions 5452 to 6840). Identical CVA-TA genome sequences were obtained from the three replicates. The CVA-TA isolate shared 98% and 81% nucleotide identity with two previously reported CVA genomes, those with accession numbers X82547 and FN691959, respectively.

The 106-nt CVA-TA 5′-UTR is 52 nt longer than those from X82547 and FN691959. A comparison of the predicted secondary structures (with lowest free energy) of 5′-UTRs from CVA-TA and X82547/FN691959 indicated that the extra 5′-UTR found in CVA-TA can form additional hairpin and loop structures, which may help stabilize the whole 5′-UTR RNA. This possible function was reflected by the much lower putative *dG* values of CVA-TA 5′-UTR (-9.50) than those from CVA isolates X82547 and FN691959 (-1.50). The extra 5′-UTR region in CVA-TA may be added during the evolution of the CVA genome in response to local environmental signals.

### Nucleotide sequence accession number.

The whole-genome sequence of CVA-TA has been deposited in GenBank under the accession no. KU131205.
